# A study on the association between prefrontal functional connectivity and non-suicidal self-injury in adolescents with depression

**DOI:** 10.3389/fneur.2024.1382136

**Published:** 2024-04-22

**Authors:** Yan Guo, Ruoxi Lu, Yiwen Ou, Yuxin Huang, Jianyu Li, Ying Cui, Danian Li, Yanting Zheng, Xinyu Liang, Shijun Qiu, Yujie Liu

**Affiliations:** ^1^First Clinical Medical College, Guangzhou University of Chinese Medicine, Guangzhou, Guangdong, China; ^2^State Key Laboratory of Traditional Chinese Medicine Syndrome, Guangzhou, China; ^3^Army Medical Center (Daping Hospital), Army Medical University, Chongqing, China; ^4^Department of Radiology, The First Affiliated Hospital of Guangzhou University of Chinese Medicine, Guangzhou, China; ^5^Cerebropathy Center, The Third Affiliated Hospital of Guangzhou Medical University, Guangzhou, China; ^6^Cerebropathy Center, The First Affiliated Hospital of Guangzhou University of Chinese Medicine, Guangzhou, China

**Keywords:** adolescent depression, functional magnetic resonance imaging, prefrontal cortex, functional connectivity, non-suicidal self-injurious behavior

## Abstract

**Objective:**

Among adolescents with depression, the occurrence of non-suicidal self-injury (NSSI) behavior is prevalent, constituting a high-risk factor for suicide. However, there has been limited research on the neuroimaging mechanisms underlying adolescent depression and NSSI behavior, and the potential association between the two remains unclear. Therefore, this study aims to investigate the alterations in functional connectivity (FC) of the regions in the prefrontal cortex with the whole brain, and elucidates the relationship between these alterations and NSSI behavior in adolescents with depression.

**Methods:**

A total of 68 participants were included in this study, including 35 adolescents with depression and 33 healthy controls. All participants underwent assessments using the 17-item Hamilton Depression Rating Scale (17-HAMD) and the Ottawa Self-Harm Inventory. In addition, functional magnetic resonance imaging (fMRI) data of the participants’ brains were collected. Subsequently, the FCs of the regions in the prefrontal cortex with the whole brain was calculated. The FCs showing significant differences were then subjected to correlation analyses with 17-HAMD scores and NSSI behavior scores.

**Result:**

Compared to the healthy control group, the adolescent depression group exhibited decreased FCs in several regions, including the right frontal eye field, left dorsolateral prefrontal cortex, right orbitofrontal cortex, left insula and right anterior cingulate coetex. The 17-HAMD score was positively correlated with the frequency of NSSI behavior within 1 year (r_s_ = 0.461, *p* = 0.005). The FC between the right anterior cingulate cortex and the right precuneus showed a negative correlation with the 17-HAMD scores (r_s_ = −0.401, *p* = 0.023). Additionally, the FC between the right orbitofrontal cortex and the right insula, demonstrated a negative correlation with the frequency of NSSI behavior within 1 year (r_s_ = −0.438, *p* = 0.012, respectively).

**Conclusion:**

Adolescents with depression showed decreased FCs of the prefrontal cortex with multiple brain regions, and some of these FCs were associated with the NSSI frequency within 1 year. This study provided neuroimaging evidence for the neurophysiological mechanisms underlying adolescent depression and its comorbidity with NSSI behavior.

## 1 Introduction

Depression is a common clinical psychiatric disorder characterized by long-term and significant depressive mood, anhedonia, and feelings of fatigue. With the acceleration of social pace and the continuous increase in life and work pressures, the prevalence of depression has been steadily rising across all age groups. According to statistics, the increase in prevalence among adolescents has even surpassed that of adults, with a lifetime prevalence rate of up to 11% (1). Adolescents with depression often exhibit poor emotional stability, impulsivity, self-criticism, and negative emotions, while lacking effective emotional regulation abilities. Consequently, self-harm and self-injury may become a means for them to regulate emotions and alleviate stress (2).

Non-suicidal self-injury (NSSI) refers to the deliberate and repetitive act of self-harming without the intention of suicide (2). However, the presence of NSSI in adolescents with depression increases their risk of developing suicidal thoughts or engaging in suicidal behavior by more than 20 times (3). Previous studies have shown that NSSI behavior in adolescents with depression is associated with cognitive deficits and emotional dysregulation (4, 5), as well as more severe impairments in decision-making (6). Unlike adults, adolescents often do not seek help for their mental health issues. Therefore, addressing the mental health problems of adolescents has become an undeniable societal challenge. There is an urgent need for simple, non-invasive, and reliable scientific techniques to investigate the biological mechanisms of adolescent depression and NSSI behavior. This would aid in early and accurate diagnosis of the disorder and facilitate the implementation of effective and proactive treatment approaches.

In recent years, there has been a growing interest in investigating the pathogenesis of adolescent depression using functional magnetic resonance imaging (fMRI). Previous studies showed that the prefrontal cortex may play a crucial role in regulating emotional responses (7), and abnormal functional connectivity (FC) in this brain region may be closely related to NSSI behavior (8). However, limited research has been conducted on the correlation between altered FC in the prefrontal cortex and NSSI among adolescents with depression. Therefore, this study focuses on drug-naïve adolescents aged 12–18 years who are experiencing their first episode of depression. The primary objective of this study is to investigate the alterations in FC of the regions in the prefrontal cortex with the whole brain in adolescents with depression, and elucidates the relationship between these alterations and NSSI behavior in adolescents with depression. By gaining a better understanding of the underlying mechanisms of comorbid depression and NSSI in adolescents, this research aims to contribute to early prediction and intervention strategies.

## 2 Methods and materials

### 2.1 Participants

A total of 35 adolescents with depression and 33 healthy controls (HCs) were included in this study. Adolescents with depression were recruited from the psychological counseling outpatient clinic of the First Affiliated Hospital of Guangzhou University of Chinese Medicine from September 2019 to September 2020. The diagnosis of treatment-naïve, first-episode depression was made by two attending psychiatrists, each of whom had more than 10 years of experience in depression diagnosis. The Diagnostic and Statistical Manual of Mental Disorders (DSM)-5 and the Structured Clinical Interview for the DSM (SCID) was used to assess whether the diagnostic criteria were met. The 17-item Hamilton Depression Rating Scale (HDRS-17) was used to evaluate the severity of depression. Each patient self-reported a rough estimate of illness duration. The other inclusion criteria for adolescents with depression were as follows: (1) aged between 12 and 18 years old, (2) HDRS-17 score > 17, (3) right-handed native Chinese speaker, and (4) free of any history of neurological illness or any other psychiatric disorder according to the DSM-5. Exclusion criteria included (1) a history of any significant illness, (2) contraindications to MRI scans. The HCs were all volunteers who were physically healthy based on their self-reported medical history and mentally healthy according to the Mini-International Neuropsychiatric Interview (MINI) as applied by two psychologists. Besides, the HDRS-17 score of HCs was <7. This study was conducted in accordance with the Declaration of Helsinki. All participants and their legal guardians provided written informed consent, and the study was approved by the Ethics Committee of the First Affiliated Hospital of Guangzhou University of Chinese Medicine, Guangzhou, China.

### 2.2 Ottawa Self-Injury Inventory testing

The Ottawa Self-Injury Inventory (OSI) is a self-assessment tool that serves as a comprehensive reporting instrument for evaluating NSSI behavior. It enables a thorough assessment by evaluating the actual frequency of engaging in NSSI behavior. Additionally, the questionnaire collects information about the methods and specific anatomical sites used for self-injury. It also explores the role of NSSI behavior in the release of negative emotions, the means through which individuals acquire knowledge about NSSI, strategies employed for preventing NSSI occurrences, and seeking help and treatment after engaging in NSSI behaviors. By encompassing these key elements, the OSI enables a comprehensive evaluation of NSSI behavior and related aspects, aiding in a better understanding of the condition and informing interventions. Besides, we excluded subjects with suicidal thoughts and behaviors based on question 4 of the OSI to minimize confounding effects of clinical heterogeneity as much as possible.

### 2.3 Image acquisition

Data were acquired on a Siemens MAGNETOM Prisma 3.0 T MRI scanner (Siemens, Erlangen, Germany) using a 64-channel radiofrequency head coil and the Siemens SMS BOLD [two-dimensional (2D) multiband gradient echo EPI (SMS-EPI)] sequence within 3 days of diagnosis. The participants were instructed to close their eyes and refrain from thinking anything. Two radiologists made consensus decisions that all participants were free of visible brain abnormalities or any form of lesions based on thick-slice axial T1-and T2-weighted images as well as T2-weighted fluid-attenuated inversion recovery (T2-FLAIR) images. The parameters of rs-fMRI included TR = 500 ms, TE = 30 ms, matrix size = 64 × 64, no slice gap, field of view (FOV) = 224 mm × 224 mm, slice thickness = 3.5 mm, slice number = 35, 960 time points. The parameters of three-dimensional T1-weighted images (3D-T1WI) included slice thickness = 1 mm, no slice gap, FOV = 256 mm × 256 mm, TR = 2,530 ms, TE = 2.98 ms, flip angle = 7°, and 192 slices.

### 2.4 Image preprocessing

Image preprocessing was performed using SPM12^1^ and DPARSF version 2.3.^2^ The images were corrected for acquisition time intervals between slices and head motion between volumes. No data was discarded since no the participant’s maximum cumulative head motions exceeded 1.5 mm in translation or 1.5° in rotation along any direction. Next, 3D-T1WI data were coregistered to the fMRI data of the same subject and further segmented using unified segment (see text footnote 1) and registered to the standard Montreal Neurological Institutes (MNI) space using diffeomorphic anatomical registration through exponentiated Lie algebra (DARTEL). The rs-fMRI data were then warped to MNI space according to the generated deformation field and smoothed with a Gaussian kernel of 6 mm full width at half maximum (FWHM). Several nuisance signals, including the Friston-24 head motion parameters and mean signals from cerebrospinal fluid and white matter, were regressed out from the fMRI data. Then, linear detrending and bandpass filtering (0.01–0.08 Hz) were performed to reduce low-frequency drift and high-frequency noise.

### 2.5 FC analysis

In this study, various regions of the prefrontal cortex were selected as region of interests (ROIs) for FC analysis. These ROIs were defined based on Brodmann template, specifically Brodmann areas 8–13, 24/32, and 44–47, which were categorized as parts of the prefrontal cortex (9). Firstly, the WFU_pickatlas toolbox was used to extract bilateral regions 8, 9/46, 10, 11/12, 13, 24/32, 44, 45, and 47 as masks, based on the Brodmann template. The voxel size of the generated masks was 1 mm × 1 mm × 1 mm, which was later resliced to 3 mm × 3 mm × 3 mm using the Reslice Image function in the SPM toolbox. Using DPARSF version 2.3, we computed Pearson correlation coefficients between the mean time series of each ROI and that of each voxel of the whole brain. Then, a Fisher r-to-z transformation was used to convert the correlation coefficient to z values to improve normality. Finally, we obtained z-score of the FC maps of each individual for further analysis. Next, we used SPM 12 (see text footnote 1) to perform two-sample t-tests (gender, age, and education as covariates) to determine areas with significantly different FCs to the ROIs between adolescents with depression and HCs. We used *p* < 0.001 for the cluster-forming threshold and implemented a family-wise error (FWE) correction approach at the cluster level. All results survived whole-brain cluster correction (*P*_FWE_ < 0.05).

### 2.6 Analysis of clinical data

IBM SPSS Statistics 26.0 was utilized to perform statistical analysis on various factors including age, years of education, BMI, 17-HAMD scores, disease duration, and the number of NSSI behaviors for both the depressed adolescent group and the HC group. The normality of the data was tested using the Shapiro–Wilk test. For normally distributed and homogeneous data, descriptive statistics (mean ± standard deviation,^−^x ± s) were used, and group comparisons were conducted with independent samples t-tests. For data that did not meet the assumptions of normality, medians with corresponding quartiles M (P25–P75) were used, and group comparisons were performed using non-parametric tests (Mann–Whitney U-test). Gender distribution between the depressed adolescent group and the HC group was presented as frequencies, and a Pearson chi-square test was conducted for group comparisons. *p* < 0.05 was considered statistically significant.

### 2.7 Statistical analysis

The statistical analysis in this study was mainly conducted on the MATLAB_R2013b platform, using the SPM12 toolbox for data analysis. Two independent sample t-tests were performed on the FCs of the depressed adolescent group and the HC group. Age, gender, years of education and gray matter maps were utilized as covariates. The analysis involved family-wise error (FWE) correction with a significance level set at a single voxel *p* < 0.01, using a two-tailed test. For the identified FCs showing differences between the two groups, the xjview toolbox^3^ was used for anatomical localization and visualization of the structures. The location, voxel size, peak MNI coordinate, and statistical t-value of the brain regions with statistically significant differences were extracted. Next, the xjview toolbox was utilized to define these ROIs for further analysis. The REST_PLUS toolbox^4^ was employed to extract the FCs between the statistically significant brain regions in the two groups for subsequent analysis.

Rank correlation analysis was conducted to explore the relationships between the values of the statistically significant brain regions, disease severity, disease duration, and the NSSI frequency. A *p* < 0.05 (two-tailed) was considered statistically significant in these correlation analyses. Gender comparisons between the two groups were assessed using Pearson’s chi-square test, with a *p* < 0.05 considered statistically significant.

## 3 Results

### 3.1 Demographic and clinical characteristics

This study involved a total of 68 participants, with 35 individuals in the adolescent depression group (21 females, 14 males) and 33 individuals in the HC group (16 females, 17 males). No significant difference was found between the adolescent depression group and the HC group in terms of age, gender, education, and BMI. The 17-HAMD scores in the adolescent depression group were significantly higher than those observed in the HC group, with a statistically significant difference (*p <* 0.05; Table 1).

**Table 1:** Demographic characteristics and NSSI scores of the participants.

	adolescent depression (*n* = 35)	HC (*n* = 33)	z/χ2	*P-*value
Age, yrs	15 (14~16)	16 (14.5~17)	−1.812	0.070^†^
Gender (F/M)	14/21	17/16	0.908	0.341^‡^
Education (yrs.)	9 (8~11)	10 (8.5~12)	−1.476	0.140^†^
17-HAMD score	20 (18~24)	4 (2.5~5)	17.289	<0.001^†^
BMI	18.59 (17.21~20.20)	19.15 (17.37~21.11)	−0.736	0.462^†^
Illness duration (months)	24 (12~36)	N/A	N/A	N/A
NSSI frequency (1 month)	1 (1~4)	N/A	N/A	N/A
NSSI frequency (6 months)	4 (3~6)	N/A	N/A	N/A
NSSI frequency (1 yrs.)	10 (6–23)	N/A	N/A	N/A

HC, healthy control; 17-HAMD, 17-item Hamilton Depression Rating Scale; BMI, Body Mass Index; NSSI, Non-Suicidal Self-Injury. Age, education, 17-HAMD score, BMI, illness duration, and NSSI frequency (the past 1 and 6 months, the past 1 years) did not follow a normal distribution and were described in terms of the median and interquartile range (P25–P75). ^†^The *p*-values were obtained by Mann–Whitney test. ^‡^The *p*-value was obtained by a chi-squared test.

**Figure 1:**
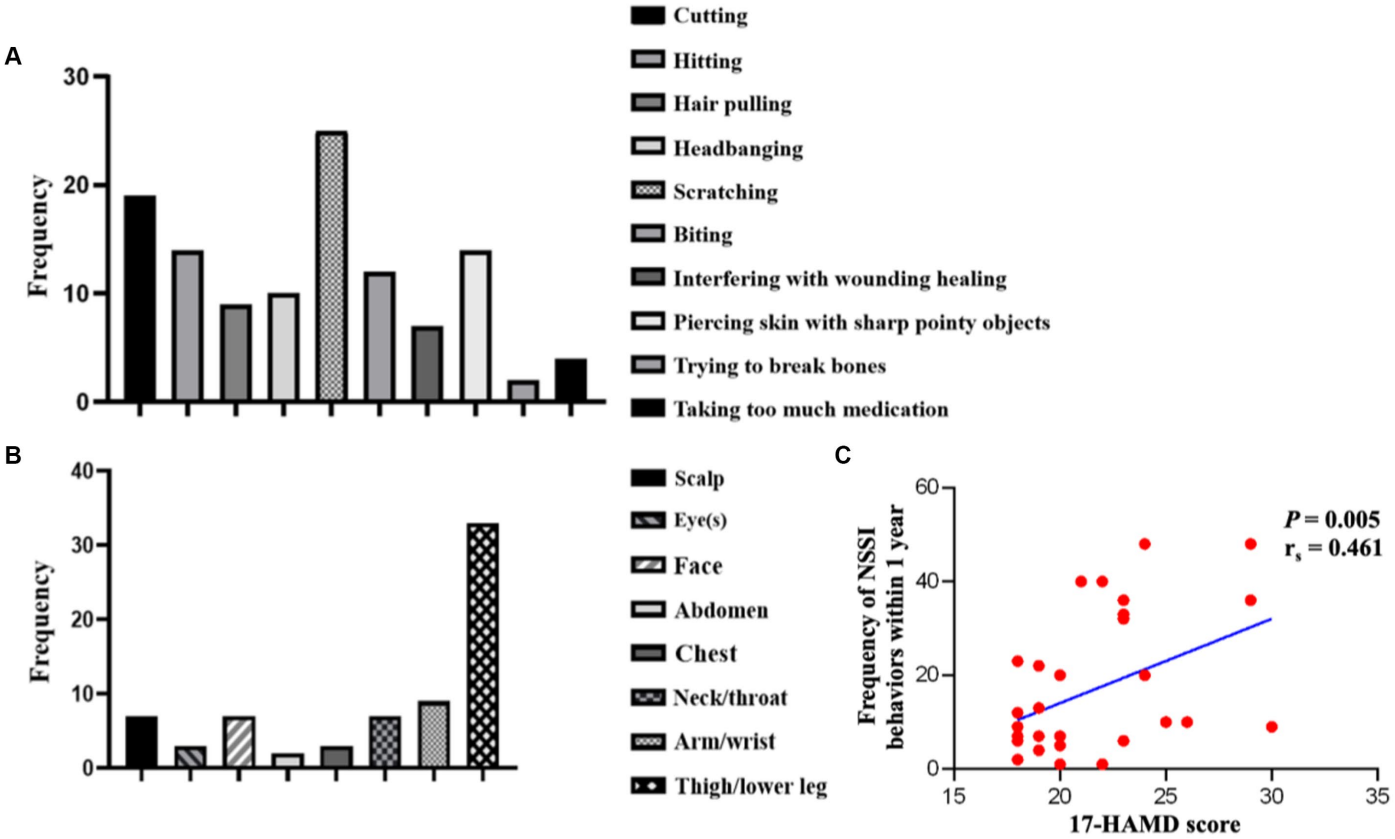
Methods and sites of NSSI behaviors, and correlation between 17-HAMD scores and NSSI frequency in the adolescent depression group. NSSI, non-suicidal self-injury. **(A)** Methods and Sites of NSSI behaviors. **(B)** Sites of NSSI behaviors. **(C)** Correlation between 17-HAMD scores and the frequency of NSSI behaviors within 1 year.

The statistical analysis of the NSSI in the adolescent depression group revealed that these patients engage in NSSI behavior an average of more than 10 times in the past year. The common methods of implementation included scratching (25/35), cutting (19/35), hitting (14/35), piercing skin with sharp pointy objects (14/35), biting (12/35), headbanging (10/35), hair pulling (9/35), interfering with wounding healing (7/35), taking too much medication (4/35), and trying to break bones (2/35; Figure 1A). The most common NSSI behavior was scratching. The common sites of self-injury included arm/wrist (33/35), legs (9/35), scalp (7/35), face (7/35), neck (7/35), eyes (3/35), abdomen (3/35), and chest (2/35), with arm/wrist being the most common site of self-injury (Figure 1B). A positive correlation was found between the 17-HAMD score and the frequency of NSSI behaviors within 1 year in the adolescent depression group (*r_s_* = 0.461, *p* = 0.005, Figure 1C).

### 3.2 Differences in FCs between the adolescent depression group and the HC group

This study found statistically significant differences in whole-brain FCs between the adolescent depression group and the HC group in the five ROIs (*P_FWE_* < 0.05). See Table 2 for details.

1. The bilateral frontal eye fields

**Table 2:** Brain regions showing FC changes in adolescents with depression using the ROIs of the prefrontal cortex.

	ROI	Cluster	Brain region	Size	Peak MNI coordinates			T-value
					X	Y	Z	
Adolescent with Depression< HC								
	BA 8 (Eye Field_R)	1	Occipital_Mid_R	244	33	−51	30	3.8866
	BA 9/46 (DLPFC_L)	2	Frontal_Inf_Tri_L	206	−36	45	−9	3.9095
	BA 11/12 (Orbitofrontal area_R)	3	Temporal_Mid_R	156	39	27	42	3.9033
		4	Insula_R	259	36	30	0	4.2191
		5	Occipital_Mid_R	165	36	−78	30	3.9021
	BA 13 (Insula_L)	6	Calcarine_L	205	−18	−99	−6	3.6676
	BA 24/32 (ACC_R)	7	Calcarine_L	205	−18	−99	16	3.6676
		8	Precuneus_R	170	6	−69	45	3.8938

ROI, region of interest; Eye Field_R, right frontal eye field; DLPFC_L, left dorsolateral prefrontal cortex; Orbitofrontal area_R, right orbitofrontal cortex; Insula_L, left insula; Insula_R, right insula; ACC_R, right anterior cingulate cortex; Occipital_Mid_R, right middle occipital gyrus; Frontal_Inf_Tri_L, left triangular part of the inferior frontal gyrus; Occipital_Mid_R, right middle occipital cortex; Temporal_Mid_R, right middle temporal cortex; Calcarine_L, left calcarine; Precuneus_R, right precuneus.

**Figure 2:**
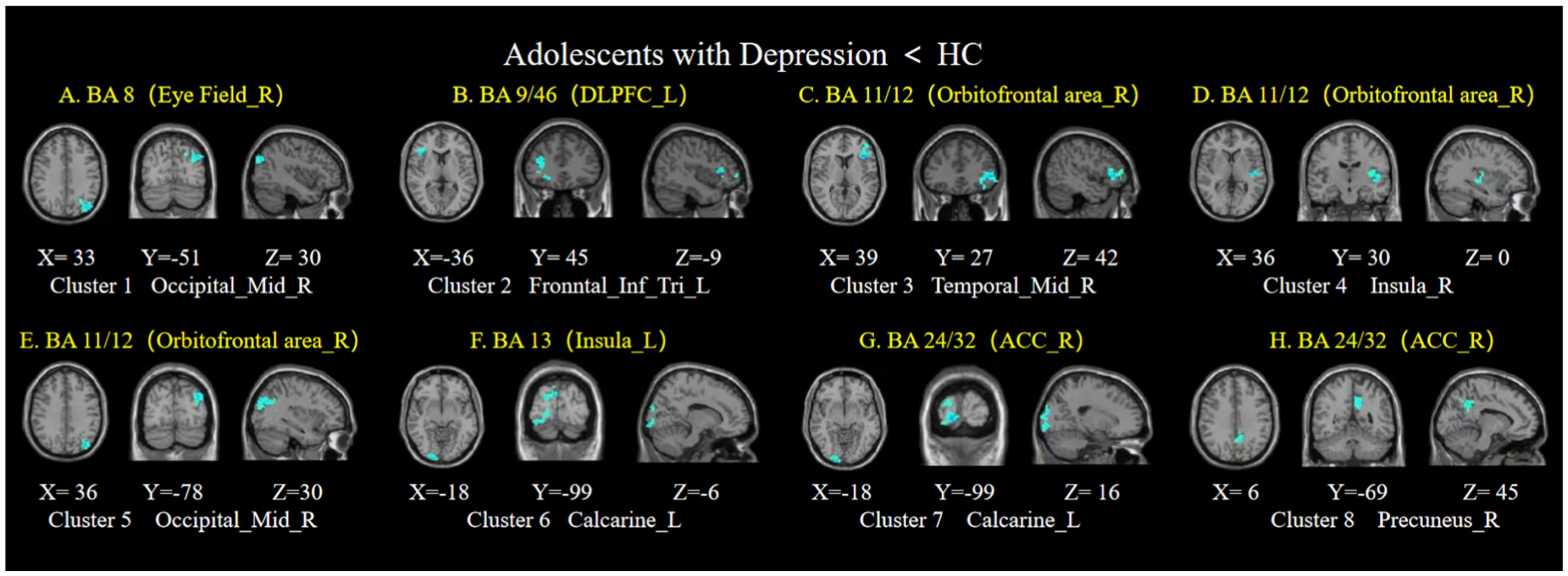
Brain regions showing FC differences based on the ROIs of prefrontal cortex. Compared to the healthy control group, the adolescent depression group showed decreased FCs between **(A)** the right frontal eye field and the right middle occipital gyrus; **(B)** the left dorsolateral prefrontal cortex and the left triangular part of the inferior frontal gyrus; **(C–E)** the right orbitofrontal cortex and the right middle temporal cortex, right insula, and right middle occipital cortex; **(F)** the left insula and the left calcarine; **(G,H)** the right anterior cingulate cortex and the left calcarine, and the right precuneus. Eye Field_R, right frontal eye field; DLPFC, dorsolateral prefrontal cortex; Orbitofrontal area_R, right orbitofrontal cortex; ACC_R, right anterior cingulate cortex; HC, healthy control.

Compared to the HC group, the adolescent depression group showed decreased FC between the right frontal eye field and the right middle occipital gyrus (Figure 2A), while no statistically significant differences were observed in the FC with the left frontal eye field.

2. The bilateral dorsolateral prefrontal cortex

Compared to the HC group, the adolescent depression group showed decreased FC between the left dorsolateral prefrontal cortex and the left triangular part of the inferior frontal gyrus (Figure 2B).

3. The bilateral orbitofrontal cortex

Compared to the HC group, decreased FCs were observed between the right orbitofrontal cortex and the right middle temporal cortex, right insula, and right middle occipital cortex (Figures 2C–E). However, no statistically significant differences were observed in the FC of the left orbitofrontal cortex in the adolescent depression group.

4. The bilateral insula

Compared to the HC group showed decreased FC between the left insula and the left calcarine (Figure 2F). No statistically significant differences were observed in the FC of the right insula in the adolescent depression group.

5. The bilateral anterior cingulate cortex

There was decreased FC between the right anterior cingulate cortex and the left calcarine (Figure 2G), as well as the right precuneus in adolescents with depression (Figure 2H). No statistically significant difference in the FC of the left anterior cingulate cortex in adolescents with depression.

### 3.3 The correlation between FCs and neuropsychological scores

**Figure 3:**
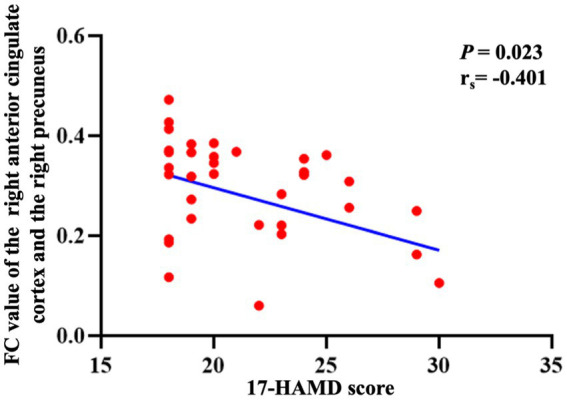
The correlation between the FC of the right anterior cingulate gyrus with the right precuneus and the 17-HAMD score. FC, functional connectivity; 17-HAMD, 17-item Hamilton Depression Rating Scale.

**Figure 4:**
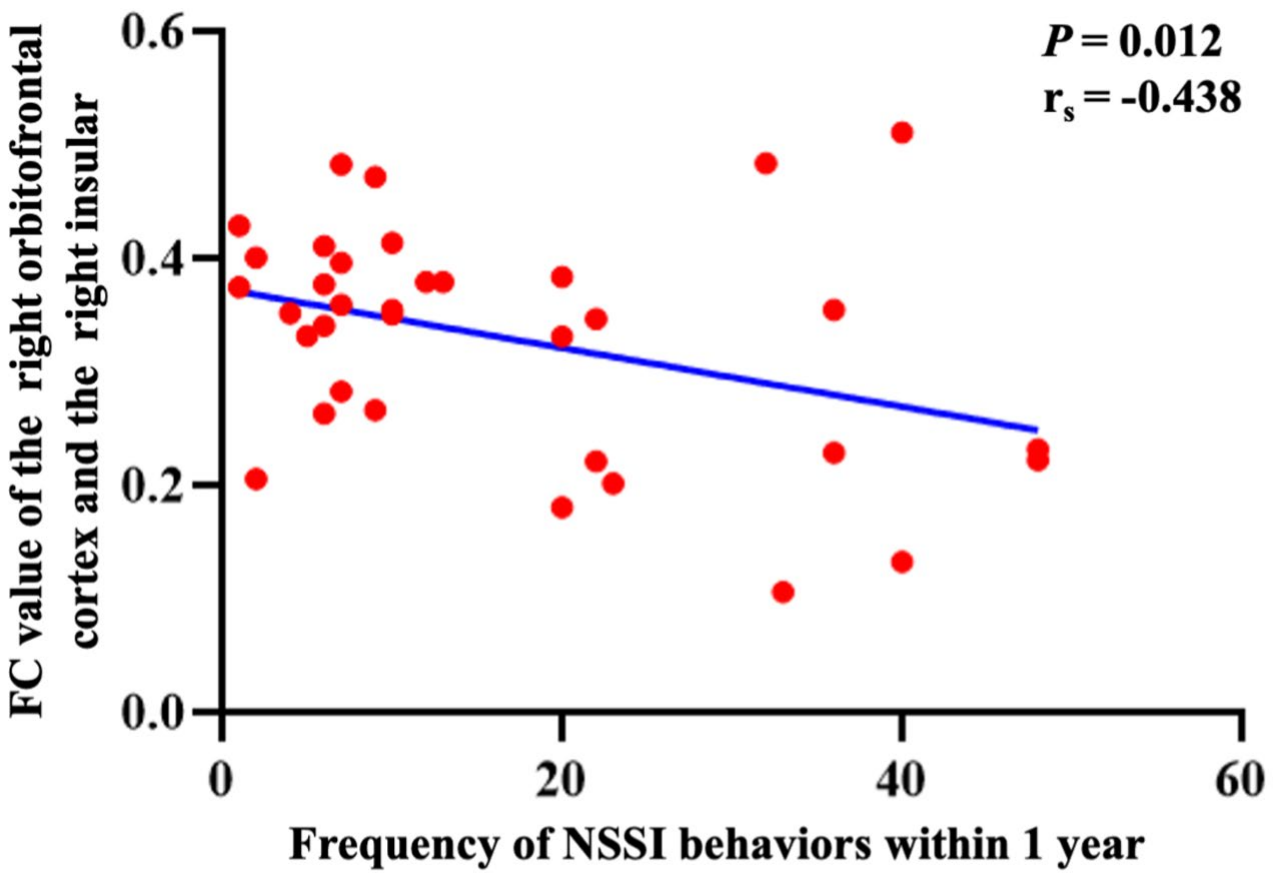
The correlation between the significant different FCs and the frequency of NSSI behaviors. The correlation between the FC of the right orbitofrontal cortex with the right insular and the frequency of NSSI behaviors within 1 year. FC, functional connectivity; NSSI, non-suicidal self-injury.

For 35 adolescents with depression, there was a negative correlation between the FC of the right anterior cingulate gyrus with the right precuneus and the 17-HAMD scores (*p* = 0.023, r_s_ = −0.401; Figure 3). The FC between the right orbitofrontal cortex and the right insular showed a negative correlation with the frequency of NSSI behavior within 1 year (*p* = 0.012, r_s_ = −0.438; Figure 4).

## 4 Discussion

In this study, we analyzed the whole-brain FC of 18 ROIs in the prefrontal cortex for 35 adolescents with depression and 33 HCs. We also examined the correlations between the FC and the 17-HAMD scores, as well as the frequency of NSSI behaviors within 1 year. The results of this study indicated that in adolescents with depression, there was a positive correlation between the 17-HAMD scores and the frequency of NSSI behaviors within 1 year. There were significant differences in the FCs of five ROIs between the two groups. Specifically, there was a negative correlation between the frequency of NSSI behaviors within 1 year and the FC values of the right orbitofrontal cortex with the right insular. This study provides neuroimaging evidence for the neurophysiological mechanisms underlying adolescent depression and its comorbidity with NSSI behavior.

### 4.1 Whole-brain FC analysis of prefrontal cortex in adolescents with depression

This study found abnormal FC between the prefrontal cortex and multiple brain regions in adolescents with depression, including the frontal, parietal, and occipital cortex. The results suggested that depression is a neuropsychiatric disorder with high heterogeneity and complex pathophysiology.

This study found decreased FC between the right frontal eye field and the right middle occipital gyrus in the adolescents with depression. Previous studies have indicated that various emotional disorders, such as depression and anxiety, often involve attention biases toward emotional visual stimuli (10). Individuals with these disorders tend to focus more on negative stimuli, rather than pleasant stimuli (11). In our study, the frontal eye field involved in the visual conduction pathway, was dysfunctional in the adolescent depression group. This is consistent with previous findings, such as Feldmann et al. (12) conducted two eye-tracking studies, indicating that adolescents with depression have lower cognitive evaluation of negative emotional pictures compared to the HC group, suggesting an association among depression, the abnormalities in emotional regulation and visual attention processes.

This study also found that FC in multiple brain regions involved in emotion regulation (e.g., the dorsolateral prefrontal cortex, the insula, the anterior cingulate cortex (ACC), the orbitofrontal cortex, etc.) decreased. Previous neuroimaging study have suggested a close association between the dorsolateral prefrontal cortex and the neurobiology of depression, and it is a common target for repetitive transcranial magnetic stimulation in the treatment (13). Biermannd et al. (14) found an imbalance between cortical excitation and inhibition in the dorsolateral prefrontal cortex of adolescents with depression using transcranial magnetic stimulation-synchronized electroencephalogram, and this excessive inhibition in the dorsolateral prefrontal cortex was associated with depressive symptoms in adolescents, which aligns with the findings of decreased FC in the dorsolateral prefrontal cortex observed in this study. The inferior frontal gyrus is mainly responsible for various functions related to language, internal perception, and emotional information processing (15, 16). A study found decreased FC in the left triangular part of the inferior frontal gyrus in depressed patients compared to HCs (17). The insula has extensive connections with surrounding structures and is adjacent to several important brain structures (18). It participates in the constituting of the salience network, thus playing a crucial role in brain function and being involved in various complex functions (19). In a study by Hall et al. (20), whole-brain analysis revealed lower activation levels in the right insula of adolescents with depression compared to the HC group. The ACC has been shown to be a critical brain region involved in depression (21–23). In this study, we observed a reduction in the FC between the ACC and the precuneus, indicating potential disruptions in the neural pathways related to emotional processing in adolescent depression. Previous research has found decreased FC in the ACC neural network in adolescents with depression (24).

The above findings confirmed the results of previous studies, however, our study yielded some different results. Specifically, we uncovered a decreased FC between the right orbitofrontal cortex (OFC) and the right insula. In contrast, a longitudinal study conducted by Jin et al. (25) on 229 adolescent females without a history of depression revealed an increased FC between the OFC and posterior insula. This contradicts the decreased FC observed in our study. Potential reasons for this inconsistency may stem from differences in sample selection and duration of depressive symptoms. Consequently, further longitudinal research with larger sample sizes and extended durations is imperative to elucidate the underlying mechanisms of OFC in the development of depression.

### 4.2 Analysis of the correlation among abnormal FC, 17-HAMD scores and NSSI frequency

The 17-HAMD scale is commonly used to assess the severity of depressive symptoms, with higher scores indicating more severe depressive emotions. This study found a positive correlation between the 17-HAMD scores and the frequency of NSSI behaviors within 1 year. This suggests that as the severity of depressive symptoms increases, the frequency of NSSI behaviors also tends to increase. Additionally, the study found a negative correlation between FC values of the right ACC with the right precuneus and 17-HAMD scores. ACC plays a key role in the processing of emotions and is an integral part of various systems including emotion, sensation, attention, and working memory (26). The decreased FC between the ACC and precuneus suggests poor emotion regulation and reduced ability to regulate negative emotions in adolescents with depression. Moreover, as the severity of depressive symptoms increases, the FC of these brain regions becomes weaker. These findings indicate that the degree of FC changes between the right ACC and right precuneus could serve as a biomarker for assessing the severity of depressive symptoms in the depressed group.

Patients in the adolescent depression group have poor emotional stability, strong impulsivity, lack of effective emotion regulation, and are prone to self-injurious behavior as a way to regulate emotions and relieve stress (2), this pattern of behavior is reinforced by the temporary satisfaction gained from repeated self-injurious behaviors. In addition, impaired emotional regulation leads to an increase in NSSI in adolescents (27). The higher the degree of emotional dysregulation, the higher the risk of developing NSSI (28). According to the emotion-cognitive regulation model (29), individuals may alleviate negative emotions by seeking negative stimuli and reward-sensitive, resulting in NSSI behaviors. Previous studies have suggested that overactivation of brain regions related to the reward circuit affects adolescent risk reward processing in decision-making, showing stimulus-seeking and reward-sensitive, from NSSI behavior (30, 31). In this study, we found that decreased FC between the OFC and the insula was negatively associated with the frequency of the NSSI behaviors within 1 year. Previous studies have only reported a noticeable decrease in insula volume in adolescents with NSSI (32). The decreased FC between the OFC and the insula, previously unreported in the literature, was a significant finding. The OFC and the insula were part of the brain’s reward circuit, which were involved in risk decision-making and pain perception. NSSI individuals have been shown to have higher pain thresholds and tolerances (33), with lower perceived pain intensity (34, 35). Therefore, we hypothesize that the decreased FC between the OFC and insula may affect reward processing in adolescent risk decision-making, resulting in stimulus-seeking behavior and heightened sensitivity to rewards, ultimately leading to NSSI.

### 4.3 Limitations of this study

This study has some limitations. Firstly, this study was cross-sectional and lacked longitudinal follow-up observations. The sample size of 68 cases was relatively small, and the study did not further investigate adolescents with depression who did or did not engage in NSSI behaviors. Therefore, our analysis was limited to investigating the correlation between NSSI frequency and FC. It is evident that NSSI is influenced by multiple factors, necessitating a comprehensive examination of clinical, emotional, and behavioral elements. Future studies should address NSSI as a multifactorial construct, understanding the underlying mechanisms and risk factors contributing to NSSI engagement, emphasizing early prevention strategies to mitigate harm, and conducting longitudinal follow-up studies to further explore the mechanisms underlying adolescent depression and NSSI behaviors. Moreover, the short TR time (500 ms) used in fMRI data acquisition in this study can significantly enhance spatial resolution but may also impact the signal-to-noise ratio, thus affecting experimental efficacy. Previous studies have emphasized the importance of optimizing temporal resolution in small-scale studies, with a focus on signal-to-noise ratio, experimental power, and reliability (36). As a result, in the future, we plan to further increase the sample size and consider using the TR time of 2,000 ms to gather fMRI data. Additionally, in effort to further validate the reproducibility of our findings, we reprocessed our raw data using the fMRIPrep tool^5^ and applied the ICA-AROMA method for automatic denoising. Despite achieving some reproducible outcomes, they did not withstand correction for multiple comparisons. We speculate that the AROMA method may have removed a significant amount of useful signal, resulting in negative results. As a solution, we plan to expand the sample size and utilize AROMA for automatic fMRI denoising to minimize the impact of noise while preserving valuable signal, thus improving the study’s reproducibility and reliability. Lastly, the various regions of the prefrontal cortex have different functions, and each region can be further subdivided into finer subregions. The use of the classic Brodmann areas in this study, although well-established, may result in larger divisions of the prefrontal cortex. Therefore, future research could consider further subdividing the different regions of the prefrontal cortex, using more refined brain functional templates to explore the role of the prefrontal cortex in the neurophysiological mechanisms of adolescent depression.

## 5 Conclusion

Adolescents with depression showed decreased FCs of the prefrontal cortex with multiple brain regions, and some of these FCs were associated with the frequency of NSSI behaviors within 1 year. This study provided neuroimaging evidence for the neurophysiological mechanisms underlying adolescent depression and its comorbidity with NSSI behavior.

## Data availability statement

The raw data supporting the conclusions of this article will be made available by the authors, without undue reservation.

## Ethics statement

The studies involving humans were approved by the Ethics Committee of the First Affiliated Hospital of Guangzhou University of Chinese Medicine, Guangzhou, China. The studies were conducted in accordance with the local legislation and institutional requirements. Written informed consent for participation in this study was provided by the participants’ legal guardians/next of kin.

## Author contributions

YG: Conceptualization, Investigation, Methodology, Writing – original draft, Data curation, Formal analysis, Software. RL: Formal analysis, Investigation, Methodology, Software, Writing – original draft. YO: Formal analysis, Writing – original draft, Data curation. YH: Data curation, Formal analysis, Writing – original draft. JL: Data curation, Writing – original draft, Methodology. YC: Data curation, Methodology, Writing – original draft. DL: Data curation, Methodology, Writing – original draft. YZ: Data curation, Validation, Writing – original draft. XL: Supervision, Validation, Writing – original draft. SQ: Funding acquisition, Methodology, Project administration, Supervision, Writing – review & editing. YL: Conceptualization, Funding acquisition, Investigation, Methodology, Project administration, Supervision, Validation, Writing – original draft, Writing – review & editing.

## Footnotes

^1^ www.fil.ion.ucl.ac.uk/spm

^2^ http://rfmri.org/DPARSF

^3^ https://www.alivelearn.net/xjview

^4^ http://www.restfmri.net/forum/restplus

^5^ http://www.nipreps.org
